# Gating and Regulation of KCNQ1 and KCNQ1 + KCNE1 Channel Complexes

**DOI:** 10.3389/fphys.2020.00504

**Published:** 2020-06-04

**Authors:** Yundi Wang, Jodene Eldstrom, David Fedida

**Affiliations:** Department of Anesthesiology, Pharmacology & Therapeutics, The University of British Columbia, Vancouver, BC, Canada

**Keywords:** KCNE1, KCNQ1, IKs, activation gating, calmodulin, single channels, PKA

## Abstract

The IKs channel complex is formed by the co-assembly of Kv7.1 (KCNQ1), a voltage-gated potassium channel, with its β-subunit, KCNE1 and the association of numerous accessory regulatory molecules such as PIP2, calmodulin, and yotiao. As a result, the IKs potassium current shows kinetic and regulatory flexibility, which not only allows IKs to fulfill physiological roles as disparate as cardiac repolarization and the maintenance of endolymph K^+^ homeostasis, but also to cause significant disease when it malfunctions. Here, we review new areas of understanding in the assembly, kinetics of activation and inactivation, voltage-sensor pore coupling, unitary events and regulation of this important ion channel complex, all of which have been given further impetus by the recent solution of cryo-EM structural representations of KCNQ1 alone and KCNQ1+KCNE3. Recently, the stoichiometric ratio of KCNE1 to KCNQ1 subunits has been confirmed to be variable up to a ratio of 4:4, rather than fixed at 2:4, and we will review the results and new methodologies that support this conclusion. Significant advances have been made in understanding differences between KCNQ1 and IKs gating using voltage clamp fluorimetry and mutational analysis to illuminate voltage sensor activation and inactivation, and the relationship between voltage sensor translation and pore domain opening. We now understand that the KCNQ1 pore can open with different permeabilities and conductance when the voltage sensor is in partially or fully activated positions, and the ability to make robust single channel recordings from IKs channels has also revealed the complicated pore subconductance architecture during these opening steps, during inactivation, and regulation by 1−4 associated KCNE1 subunits. Experiments placing mutations into individual voltage sensors to drastically change voltage dependence or prevent their movement altogether have demonstrated that the activation of KCNQ1 alone and IKs can best be explained using allosteric models of channel gating. Finally, we discuss how the intrinsic gating properties of KCNQ1 and IKs are highly modulated through the impact of intracellular signaling molecules and co-factors such as PIP2, protein kinase A, calmodulin and ATP, all of which modulate IKs current kinetics and contribute to diverse IKs channel complex function.

## The World of Kv7.1 and Its Accessory Subunits

Potassium ion channels are categorized structurally and functionally into four major classes; calcium-activated, inward rectifying, tandem two-pore domain, and voltage-gated potassium (Kv) channels, and are known to play an essential role in cell signaling in both excitable and non-excitable cells. Within the Kv7 channel family, which belongs to the voltage-gated potassium channels class, the tetrameric voltage-gated KCNQ potassium channel subfamily is comprised of five known isoforms, Kv7.1−7.5 (KCNQ1-5). Recent research has improved our understanding of the gating and regulatory mechanisms of the first isoform, Kv7.1, commonly referred to as KCNQ1, alone and in complex with its regulatory subunit KCNE1. While reviews published about 5 years ago have dealt with these topics in detail, in this review we aim to incorporate recent experiments and new insights that allow us to refine the conceptual framework established by these authors (Zaydman and Cui, 2014; Liin et al., 2015; Nakajo and Kubo, 2015; Cui, 2016).

**FIGURE 1:**
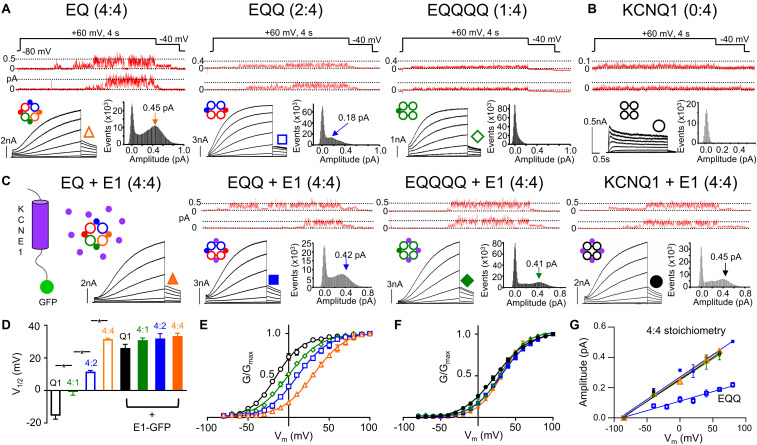
IKs channel gating depends on the stoichiometric ratio of KCNE1:KCNQ1. Representative single channel (above) and whole cell currents (below) with single channel all-points amplitude histograms of active sweeps (lower right) are shown for EQ (orange), EQQ (blue), EQQQQ (green), and KCNQ1 (black) expressed alone **(A–B)** or in combination with wild-type KCNE1-GFP (**C**, all panels). The predicted stoichiometry of KCNE1:KCNQ1 in each case is shown above. Arrows on histograms indicate the peak of the major Gaussian fit. Whole cell protocol: 4 s pulses from –80 mV to between –80 and +100 mV, followed by a repolarizing step to –40 mV for 1 s. Single channel protocol: 4 s pulses from –60 mV (–80 mV for EQQQQ) to +60 mV for 4 s, repolarized to –40 mV for 0.75 s. In A-C, topologies of expected channel Q1 and E1 complexes indicated as large hollow and small filled circles, respectively. **(D)** V_1/2_ of activation (*n* = 3–11; *p < 0.05) as indicated. Mean G-V plots from peak tail currents: KCNQ1 (black), EQQQQ (green), EQQ (blue), and EQ (orange) expressed alone **(E)** and in the presence of KCNE1 (**F**; filled symbols). **(G)** Slope conductance for each construct showing peak open amplitudes in the presence of KCNE1 (4:4 stoichiometry, EQ, EQQ, EQQQQ and KCNQ1 in the presence of KCNE1) and EQQ alone. Conductance not measured for EQQQQ or KCNQ1 alone. All error bars in this and other figures denote mean ± SE. Figure adapted from Murray et al. (2016), Figures 1–3. Reproduced with permission of CC-BY 4.0.

Expression of KCNQ1 has been detected throughout the body including in the heart, inner ear, pancreas, kidney, colon and intestine, stomach, and thyroid gland (Robbins, 2001; Liin et al., 2015; Nakajo and Kubo, 2015; Cui, 2016). When expressed alone, KCNQ1 produces a fast activating and deactivating current (Figure 1) which undergoes a mostly hidden inactivation, revealed as a hook in the tail current (Sanguinetti et al., 1996; Pusch et al., 1998; Tristani-Firouzi and Sanguinetti, 1998; Hou et al., 2017). The inactivation is quite apparent when KCNQ1 is expressed in mammalian cells (Meisel et al., 2018; Westhoff et al., 2019), but much less apparent when expressed in oocytes (Hou et al., 2017). This fast activating and deactivating KCNQ1 current has a very low conductance (Figure 1B; Yang and Sigworth, 1998; Hou et al., 2017), but has yet to be positively identified with any endogenous current(s) in the body (Abbott, 2014). Unlike other members of the KCNQ channel subfamily, KCNQ1 has been reported to associate with all five single transmembrane domain KCNE β-subunits, KCNE1-5, which modify channel kinetics to a greater or lesser extent (Barhanin et al., 1996; Melman et al., 2002b; Eldstrom and Fedida, 2011). The sometimes dramatic modulation of KCNQ1 kinetics by specific KCNE subunits, and the expression of different KCNE subunits in various tissues to a large degree underlie the diverse physiological roles that KCNQ1 plays throughout the body (Sanguinetti, 2000). KCNE1 co-expression with KCNQ1 increases current expression and greatly slows activation and deactivation kinetics. Both KCNE2 and KCNE3 in combination with KCNQ1 induce constitutively open channels not only across, but also beyond both ends of the physiological voltage range, with KCNE3 producing currents with much larger current amplitudes (Schroeder et al., 2000b). KCNE4 and KCNE5 on the other hand have an inhibitory effect when combined with KCNQ1 and decrease current amplitude under physiological conditions (Schroeder et al., 2000a; Angelo et al., 2002; Grunnet et al., 2002).

As well as the KCNEs, KCNQ1 is also regulated by several other molecules including PIP2, ATP, calmodulin (CaM) and PKA (Loussouarn et al., 2003; Matavel and Lopes, 2009; Zaydman et al., 2013; Thompson et al., 2017) which in turn further increase the flexibility of current complexes and contribute to their importance throughout body systems. KCNE1 confers significant changes on KCNQ1 tetramer properties via a variable complex stoichiometry, and modulation of biophysical mechanisms underlying voltage gating, such as voltage sensor domain (VSD) activation, pore domain (PD) opening and VSD-PD coupling as well as mechanisms of inactivation. In addition, the roles that other modulator proteins can have at the structural interface between KCNQ1 VSD activation and the time course of PD gating further contributes to the known diversity of IKs behavior. By understanding gating mechanisms both in the presence and absence of KCNE1 we can begin to better understand the physiology of the channel complex and the pathophysiology of the various diseases associated with IKs, and also perhaps eventually develop better therapeutic treatments for patients suffering from consequential arrhythmia syndromes.

## KCNE1 Modulation of KCNQ1 Kinetics in Health and Disease

How KCNQ1 and its accessory subunit KCNE1 traffic and assemble to form complexes with unique physiological roles is still a subject of some debate. Overall, two main theories for trafficking and assembly exist, one involves KCNE1 and KCNQ1 trafficking separately, KCNE1 by a vesicular route and KCNQ1 trafficking by an endoplasmic reticulum or sarcoplasmic reticulum route (Poulsen and Klaerke, 2007; Jiang et al., 2017; Kaur et al., 2020). Once at the plasma membrane the two subunits then assemble. In this case, it has also been suggested that KCNE1 can dissociate from KCNQ1 and be replaced by either new KCNE1 or KCNE2, the latter of which has been shown to traffic independently to the cell surface (Jiang et al., 2009). Alternatively, assembly of KCNE1 and KCNQ1 occurs early during biogenesis, perhaps in the endoplasmic reticulum, following which the complex traffics together to the plasma membrane (Krumerman et al., 2004; Chandrasekhar et al., 2006), likely through the endoplasmic reticulum − plasma membrane junction and driven by KCNQ1 (Oliveras et al., 2020).

The impact of KCNE1 co-assembly on KCNQ1 is the most studied and best understood of the accessory subunits. KCNE1 co-expression depolarizes the voltage dependence of activation (G-V) of KCNQ1 by about +50 mV, slows activation 1000-fold, and delays deactivation kinetics (Sanguinetti et al., 1996), as shown in Figure 1. A fully saturated 4:4 octameric complex of KCNE1 and KCNQ1 (EQ in Figure 1A) has the slowest activation kinetics compared with KCNQ1 alone (Figure 1B) and the most positive half-activation potential (G-V V_1/2_, Figure 1E). KCNE1 increases the macroscopic current by increasing the underlying single-channel conductance and stabilizing the open pore (Werry et al., 2013; Figure 1A, upper panels, note different current scaling), while eliminating the inactivation seen in KCNQ1 channels (Tristani-Firouzi and Sanguinetti, 1998; Hou et al., 2017; Meisel et al., 2018; Figures 1A,B). These pore effects of KCNE1 are accompanied by changes in the Rb^+^/K^+^ selectivity (Pusch et al., 2000; Zaydman et al., 2014) and in the pharmacological effects of various drugs (Busch et al., 1997; Abitbol et al., 1999; Yu et al., 2013; Wrobel et al., 2016; Hou et al., 2019; Wang et al., 2020) and fatty acid analogs (Larsson et al., 2018; Liin et al., 2018).

Functionally, the co-assembly of KCNE1 with KCNQ1 reproduces most of the characteristics of the delayed rectifier potassium current, IKs, in the heart, although the occurrence of mutations in the other KCNE subunits (2−5) in clinical cases of LQTS (Eldstrom and Fedida, 2011) and lone atrial fibrillation (Olesen et al., 2014) indicate that the full exposition of IKs likely requires a more complete understanding of the contributions of KCNE subunits other than KCNE1 to the channel complex. IKs together with IKr, the hERG channel, and possible accessory subunits of its own (Abbott et al., 1999) form the main repolarizing currents of the cardiac action potential (Barhanin et al., 1996; Wang et al., 1996; Sanguinetti et al., 1996). Specifically, IKs has been reported to create a “repolarization reserve” at fast heart rates, when channels can open and current summates between beats to generate a large repolarizing current which shortens the action potential and facilitates diastolic filling of the ventricles. As KCNE1 delays activation to such a large degree, the open probability of the channel reaches only about 0.2 after 4 s at room temperature (Werry et al., 2013), which suggests that IKs channels remain 99.6% closed during the normal heartbeat (Ruscic et al., 2013) and limits their major contribution to action potential repolarization to sympathetic activation during times of stress.

Due to the critical role of IKs in heart rhythm regulation, unsurprisingly, many mutations in KCNQ1 and KCNEs have been functionally linked to life-threatening long and short QT syndromes as well as atrial fibrillation (Wang et al., 1996; Chen Y. H. et al., 2003; Moss and Kass, 2005; Olesen et al., 2014; Steffensen et al., 2015; Peng et al., 2017). IKs current has, however, also been reported in the inner ear where it plays an important role in maintaining endolymph K^+^ homeostasis. The flow of potassium ions into hair cells within the cochlea depolarizes them and results in downstream hearing transduction to the brain. Some homozygous mutations in KCNE1 have been found to cause Jervell and Lange-Nielson syndrome (JLNS), a LQT syndrome with deafness (citealpBR61; Chouabe et al., 1997). Interestingly, other homozygous LQT mutations do not seem to be associated with deafness (Jackson et al., 2014).

## Structural Properties of KCNQ1

The KCNQ1 α subunit like that of nearly all other Kv channel is comprised of six transmembrane segments, S1-S6 (Lee et al., 2009), and together four KCNQ1 monomers co-assemble as a tetramer to form the KCNQ1 channel. Transmembrane helices S1-S4 form the VSD with the S5-S6 helices forming the pore domain (PD) of the channel in a domain-swapped manner that is the VSD of one domain regulates the PD of the adjacent subunit (Long et al., 2005a; Sun and MacKinnon, 2017). The S4 segment of KCNQ1 contains a net total charge of +3 which arises from positively charged arginine residues, and this is unlike other Kv channels which contain two extra gating charges. During gating, salt bridges between a conserved glutamate (E160) in S2, and positive charges in S4 (R228) present at rest, are broken and reformed with R231 and R237 (see below section on Electromechanical Coupling) as the S4 moves outward (Cui, 2016). Aqueous clefts allow extracellular or intracellular accessibility to most of these charges at rest and during channel activation as the gating charges in the S4 segment sense and move in response to changes in transmembrane voltage. The PD on the other hand contains an ion selectivity filter made up of a series of highly conserved amino acids in potassium channels, GYGDXX. In addition to the six transmembrane segments which make up the VSD and PD, the KCNQ1 monomer also contains four intracellular helices. In order to open the pore of the KCNQ1 tetramer, alone, or in complex with KCNE1, the VSD must activate, and this means that upon membrane depolarization the S4 segment moves extracellularly with some degree of rotation to transfer charges across the electric field from the inside to the outside across a charge transfer center (Tao et al., 2010). How the four VSDs within the tetramer act, whether independently, or together to open the channel pore is controversial, but it is generally understood that this translation of the S4 segment is first transferred to the S4-S5 linker. In response to this movement in the linker, the cytoplasmic lower halves of the S6 segment, which normally form a barrier to ion passage, pull apart which leads to pore opening (reviewed in Labro and Snyders, 2012). Potassium ions can then enter the selectivity filter and dehydrate, transiting the filter in coordination with backbone carbonyl oxygen atoms, along what is referred to as a “low resistance pathway” for efficient conduction of potassium ions (Doyle et al., 1998).

## Co-Assembly of KCNQ1 With KCNE1

The subunit assembly ratio of KCNE1 to KCNQ1 has been a matter of debate over the past several years, with some groups reporting a fixed KCNE1:KCNQ1 ratio of 2:4 and others reporting a variable stoichiometry between 1:4 and 4:4 depending on the concentration of KCNE1. In biochemical studies involving subunit counting and also in fluorescence photobleaching, the suggestion is that the stoichiometry is fixed at 2:4 (Chen H. et al., 2003; Morin and Kobertz, 2008; Plant et al., 2014). However, there are also suggestions that the number of KCNE1 subunits bound to KCNQ1 can be variable, based on the fact that the gating of KCNQ1 is dependent on the expression level of KCNE1 (Cui et al., 1994; Wang et al., 1998; Yu et al., 2013), the pharmacological properties (Yu et al., 2013) and other single subunit counting experiments which support a variable stoichiometry of up to 4:4 (Nakajo et al., 2010). Recent experiments using linked constructs of KCNE1 and KCNQ1 which force the stoichiometry of KCNE1 to KCNQ1 from between 1:4 to 4:4 (Figure 1) have definitively established the ability of the two proteins to assemble in different stoichiometries (Nakajo et al., 2010; Murray et al., 2016). Current activation becomes progressively slower as more KCNE1 subunits are added into the KCNQ1 ion channel tetramer compared with KCNQ1 alone (Figure 1A, right to left panels). Changes in macroscopic channel kinetics show the G-V curve of IKs becoming progressively depolarized with more KCNE1 subunits in the IKs complex (Figures 1D,E), and increased single channel conductance and latency to first opening in 4:4 compared with 1:4, 2:4, and 0:4 complexes (Murray et al., 2016; Hou et al., 2017; Figure 1A, upper panels). Co-expression of KCNE1 along with the fixed 2:4 and 1:4 constructs leads to incorporation of the free KCNE1 into spare sites in the channel complex and recapitulation of the whole cell and single channel kinetics, G-V curve and conductance of the 4:4 fully saturated heteromeric channel (Figures 1C–G). The use of targeted unnatural amino acid expression to incorporate different numbers of cross-linkable KCNE1 subunits into IKs complexes and predictably change the UV-induced current decay rate provides further strong support for the variable stoichiometry model of KCNQ1 and KCNE1 assembly (Murray et al., 2016; Westhoff et al., 2017). Interestingly, in support of these conclusions, the recent cryo-EM structure of KCNQ1+KCNE3 was captured in a 4:4 stoichiometry (Sun and MacKinnon, 2020).

Having established the potential for different numbers of KCNE1 forming complexes with KCNQ1 in oocytes and mammalian cell expression systems, the stoichiometry of association is by no means as clear physiologically in different mammals, including humans. The half-activation voltage and deactivation rates for IKs vary between dog, guinea-pig and rabbit (Chinn, 1993; Liu and Antzelevitch, 1995; Lei and Brown, 1996), all mammals used to model human cardiac electrophysiology, which suggests the possibility that different species have different KCNQ1:KCNE1 stoichiometries underlying their IKs. In human myocytes (Bosch et al., 1998; Virag et al., 2001) the kinetics, and in iPSC-CM the drug sensitivity to ML277 suggests an unsaturated ratio of 2:4 KCNE1:KCNQ1 (Yu et al., 2013), although the maturity and uniformity of iPS cells is generally not yet fully understood (Pourrier and Fedida, 2020). As well, different primary sequences for the KCNE1 subunits may contribute to the different physiological expression and kinetics of IKs in different species. An understanding of the stoichiometry of IKs is extremely important, as determining the exact numbers of regulatory subunits impacts the efficacy of pharmacological drugs, which has consequences for the treatment of cardiac arrhythmic disorders associated with mutations in KCNQ1 and KCNE1. Most activators of the IKs channel complex such as stilbenes, ML277, L-364,373 and zinc pyrithione, with the exception of mefenamic acid (Abitbol et al., 1999; Doolan et al., 2002; Magyar et al., 2006; Wang et al., 2020), phenylboronic acid (Mruk and Kobertz, 2009), and some polyunsaturated fatty acids (Larsson et al., 2018; Liin et al., 2018; Bohannon et al., 2020) are reported to have limited efficacy on both partially and fully saturated IKs complexes (Yu et al., 2013). Post-translational modification of KCNQ1 during β-adrenergic stimulation is also stoichiometrically graded with a response to cyclic adenosine monophosphate (cAMP) requiring at least one KCNE1 (Thompson et al., 2018b). A variable stoichiometry model for IKs therefore allows for great flexibility in the modulation of KCNQ1 kinetics and the IKs current.

## Evolution of Activation Models for KCNQ1 and IKs

Mechanistically, our understanding of the gating properties of voltage-dependent potassium channels such as KCNQ1 and IKs originates from analyses carried out on *Shaker* channels and the squid axon. The Markov state models used to simulate the activation of KCNQ1 and KCNQ1+KCNE1 channels, and to understand VSD involvement in pore opening, originate in the Eyring rate theory models of Eyring et al. (1949); Hodgkin and Huxley (1952). The basic assumptions of their model for the squid axon potassium channel included the idea of four identical and independent gating particles that, if positively charged, would cross the electric field from the interior of the axon to the outside before the channel could open. This led to a five-state sequential gating scheme comprising four closed states and an open state, many key features of which are still relevant almost 70 years later, not least because the cloning of potassium channels has revealed a tetrameric structure of VSDs that fits neatly into the Hodgkin-Huxley model scheme (Jan and Jan, 1989). In voltage-dependent *Shaker* channels it is generally accepted that, like in the original Hodgkin and Huxley model, the delay seen in *Shaker* channel activation requires that more than one VSD transition must occur before channel opening, and thus that all four identical VSDs must undergo independent conformational changes when the channel is closed. Unlike the original model, each VSD in the case of *Shaker* channels is preferred to undergo two independent conformational changes (resting to intermediate to activated) before the VSD becomes activated (Zagotta et al., 1994). Once all four VSDs are activated, the channel undergoes a final or multiple concerted transition(s) (Schoppa and Sigworth, 1998; Ledwell and Aldrich, 1999) from closed to open. This final transition(s) is required because channel opening after the activation delay is slower than predicted from a strict Hodgkin-Huxley model, and this makes the overall activation scheme cooperative as the opening transition depends on the state of all four subunits, which implies interaction between them (Sigworth, 1994; Zagotta et al., 1994; Horrigan et al., 1999).

These concepts from squid axon and *Shaker* potassium channels were first adapted into KCNQ1 and IKs channels by Silva and Rudy (2005). The activation delay observed suggests that the VSDs of KCNQ1 and IKs, much like those of *Shaker*, must also undergo at least two transitions during VSD activation, prior to PD opening. As such, both initial KCNQ1 and IKs models assumed a scheme where four VSDs transition independently through two conformational changes prior to full activation, and then undergo a single concerted step to allow PD opening. To simulate slow activation in IKs, the first VSD transition in the KCNQ1 model from resting to intermediate states was slowed. Both the second VSD transition rate as well as the transition between the first and second open state were further modified to be rapid. Together, these modifications ensured that more channels would remain in the resting state for a longer time and slow IKs activation. Experimentally, a delay in KCNQ1 deactivation was also seen, suggesting the presence of multiple open states between the inactivated state and the last closed state for the KCNQ1 gating model. In KCNQ1 and IKs this leads to models with 15 closed states, and different open states, and in the case of KCNQ1 alone, an inactivated state. Five open states were introduced for KCNQ1 alone, and two for IKs to account in part for activation delay and for multi-exponential deactivation kinetics. The Silva and Rudy IKs gating model was later briefly modified in 2013 to account for single channel conductance behaviors seen in IKs (Werry et al., 2013). In this model, four VSDs are postulated to independently undergo the first transition following which a sub-conducting level may be reached after at least one VSD undergoes a second independent transition to a fully activated conformation.

## Advanced Experimental Methods Give New Insight Into Activation Models

The Silva and Rudy models, like the Hodgkin and Huxley models, are classical in the sense that data from direct interrogation of VSD movement, either gating current measurements, or VCF, and from single channel measurements were not available at the time that the models were developed. In the past decade, however, data from VCF and other techniques has been collected that has improved our understanding of the underlying molecular events during KCNQ1 and IKs activation. Cysteine accessibility was initially used to show that KCNE1 and KCNE3 stabilized VSDs in the resting or active conformations, respectively, via alteration of the voltage sensor equilibrium to favor specific states (Nakajo and Kubo, 2007; Rocheleau and Kobertz, 2008). As a more direct means to follow voltage sensor movement, VCF tracks changes in emission from fluorophores attached to the extracellular end of the S4 segment as its environment changes during voltage sensing (Mannuzzu et al., 1996), and while the signals from different fluorophores can vary, it has generally been shown to give a reliable measure of S4 displacement in a variety of Kv channels (Larsson et al., 1996; Cha and Bezanilla, 1997; Claydon and Fedida, 2007; Horne et al., 2010). Gating currents provide a quantitative measure of net charge displacement during channel activation, and have supported the corresponding VCF studies in KCNQ1 alone (Ruscic et al., 2013; Barro-Soria et al., 2014). Pore dynamics and the existence of subconductance states were inferred some years ago from noise analysis and multi-channel patches (Romey et al., 1997; Sesti and Goldstein, 1998; Yang and Sigworth, 1998), but an accurate measure of IKs conductance and the ratio of KCNQ1 to IKs conductance remained elusive until the single channel kinetics were characterized more recently (Werry et al., 2013; Murray et al., 2016) and the detailed subconductance architecture of IKs channels during activation was revealed in wild-type channels, and in LQTS disease mutants (Eldstrom et al., 2015).

## Voltage Sensor Fluorescence From KCNQ1 Alone

**FIGURE 2:**
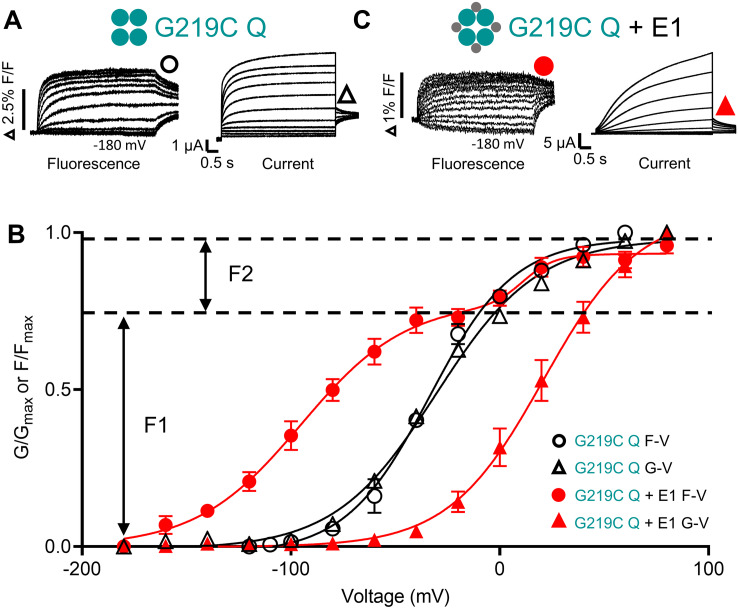
Voltage sensor movement (F-V) and pore opening (G-V) of KCNQ1 in the absence and presence of KCNE1. Fluorescence (left) and current recordings (right) are shown for G219C-KCNQ1 in the absence (G219C Q) **(A)** and presence of KCNE1 (G219C Q + E1) **(B)**. Expected channel Q1 and E1 complex topologies shown as large and small filled circles, respectively. A three-step protocol was used for current and fluorescence: 4 s pre-pulses to –140 mV (not shown), followed by 5 s steps to potentials between +80 and –180 mV, with a final repolarization step to –40 mV for 1 s. **(C)** Mean G–V and F–V plots obtained in the absence (G219C Q F-V: black circles; G219C Q G-V: black triangles) and presence of KCNE1 (G219C Q + E1 F-V: red circles; G219C Q + E1 G-V: red triangles). The G219C Q + E1 F-V was fit with a double Boltzmann function to obtain F1 and F2 components. Other F-V and G-V curves were fit with a single Boltzmann function. Fluorescence and current data were replotted from Westhoff et al. (2019), Figure 8 with permission.

Fluorescence-voltage (F-V) relationships for KCNQ1 and IKs have usually been obtained after removal of extracellular cysteines in the S3 and S6 domains, and the mutation and labeling of G219C in the S3-S4 linker with Alexa Fluor 488 C_5_-Maleimide (Alexa488) (Osteen et al., 2010; Barro-Soria et al., 2014; Hou et al., 2017; Westhoff et al., 2019). The C214A/G219C/C331A construct shows a V_1/2_ of ionic current activation that is ∼10 mV hyperpolarized to WT, but otherwise has unchanged current kinetics. Tetramethyl rhodamine (TMR) has also been used as a fluorophore, as have other locations in the S3-S4 loop, K218, and V221. A comparative study showed that these give similar, but not identical F-V relationships, with TMR exhibiting a fluorescence quenching upon depolarization, and the Alexa488 at 221C site giving the most hyperpolarized F-V relationship (Barro-Soria et al., 2014). Examples of Alexa488 fluorescence from KCNQ1 alone are shown in Figure 2A, along with ion currents. There is general consensus that when expressed without KCNE1 the fluorescence waveforms and the fluorescence-voltage relationship (F-V, representing VSD movement) of KCNQ1 overlap quite well with the time course of current activation and better with the G-V (representing pore opening) curves, respectively (Figure 2C, black symbols) (Osteen et al., 2010; Nakajo and Kubo, 2014). This overlap suggests that each VSD movement contributes in a 1:1 ratio to channel conductance (Osteen et al., 2010). In support of this conclusion that KCNQ1 channels can open when only a single VSD is activated, using the mutant I268A KCNQ1 channel, Ma et al. reported a larger than expected voltage-independent constitutive current in resting channels, which was explained by the authors as spontaneous transitions between closed and open states of the channel in the absence of any VSD movement (Ma et al., 2011). Similarly, in the mutant L353K KCNQ1 channel which locks the PD open − thus also creating a constitutive current − the VSDs are still capable of visiting a fully resting conformation (Zaydman et al., 2014). In other words, much like the mutant I268A KCNQ1 channel, the mutant L353K channel suggested that PD opening is possible with less than four, and perhaps zero activated VSDs.

Based on these experiments, a 15-state allosteric Markov gating scheme which allows PD opening and channel subconductance states even before the activation of a single VSD, was put forward to explain KCNQ1 gating (Osteen et al., 2010). No distinction was made between a model where all four VSDs moved in concert to allow PD opening, and one where VSDs moved independently, and the PD transitioned to the fully open state through subconductance levels. Although this model predicted PD opening when only one VSD was activated, and simulated experimental data well, the presence of a second voltage sensor step was not directly addressed, rather the model suggested that a second subunit movement occurred that represented pore opening to larger sublevels with each voltage sensor activated. The presence of two VSD steps during KCNQ1 channel activation as suggested in *Shaker* and IKs (Zagotta et al., 1994; Silva and Rudy, 2005) was addressed in subsequent experiments. Two fluorescence components were observed for KCNQ1 alone and interpreted to mean that the VSDs each underwent two transitions during activation as originally suggested by Silva and Rudy and analyzed in more detail in mutational studies (Wu et al., 2010; Nakajo and Kubo, 2014; Zaydman et al., 2014). Constitutive currents through both a pair of charge-reversing mutations that arrested the VSDs in either intermediate E160R/R231E (E1R/R2E) or activated E160R/R237E (E1R/R4E) states, showed that channel opening could occur when zero to four VSDs were in either intermediate or activated states. Combined with the concept that the channel can also open when all four VSDs are in the ground resting state, a 30-state allosteric model was proposed, where the VSD could either be in resting, intermediate or activated states and the PD could either be closed or open. Allosteric coupling between the voltage sensor and PD was also further decomposed in this model to its elementary components, “k” which represents PD opening and VSD activation and, “θ” which represents VSD-PD coupling.

## KCNQ1 Voltage Sensor Fluorescence in the Presence of KCNE1

There is a clear consensus that independent voltage sensor movement is allosterically coupled to PD opening in KCNQ1 channels expressed alone. When KCNQ1 is co-expressed with KCNE1, understanding activation has proven more complex. What is generally accepted in all studies is that KCNE1 dramatically increases the voltage separation between the voltage sensor fluorescence F-V and the G-V, and the fluorescence signal itself is clearly divided into two activating components, F1 and F2 (Osteen et al., 2010, 2012; Barro-Soria et al., 2014; Nakajo and Kubo, 2014; Zaydman et al., 2014; Westhoff et al., 2019). The V_1/2_ of the F1 component, which comprises about 2/3 of the fluorescence signal, is hyperpolarized to close to −100 mV in the presence of KCNE1, while the V_1/2_ of the F2 component is at ∼+20 mV, close the V_1/2_ of the G-V which is depolarized 40−50 mV in the presence of KCNE1 (Figures 2B,C). Gating charge movement shows a similar hyperpolarization when KCNQ1 is co-expressed with KCNE1, overlaying the voltage dependence of F1 and confirming that the hyperpolarized F1-V represents S4 displacement (Barro-Soria et al., 2014). It has not yet been possible to resolve gating currents that correlate with the F2 movement, and this is attributed to the slow time course of F2-dependent charge movement. However, external MTSET modification of cysteine residues placed near the top of S4 show two-step modification with depolarizations to 0 mV or above, and thus support the idea of two VSD movements during IKs activation (Barro-Soria et al., 2014). The position of the F1-V at much more negative potentials than the G-V suggests independent movement of VSDs to an intermediate state from which they do not open in the presence of KCNE1 (Zaydman et al., 2014). The voltage dependence of the second voltage sensor fluorescence component, F2, is aligned with the G-V and thus more closely associated with the activated state of the voltage sensor and pore opening.

As well as the steady-state changes, KCNE1 imposes a discordance between the time course of current and fluorescence signals, which together with the F-V to G-V separation, implies that KCNE1 induces a requirement for movement of multiple voltage sensors before channel opening (Osteen et al., 2010; Barro-Soria et al., 2014). A similar sequential gating scheme to that proposed by Silva and Rudy requiring all four VSDs to be activated prior to PD opening, with a single open state was favored. The overlap of the F2 component of the F-V curve with the G-V suggested that a concerted activation step involving all four VSDs from the intermediate to activated state occurred, after which the channel opened. In other words, the F2 component represented PD opening. This is similar to cooperative gating schemes described for *Shaker* channels, and early IKs models (Silva and Rudy, 2005) which also invoked a concerted VSD step prior to channel opening. Other groups have explained the presence of F1 and F2 components in the F-V and the wide separation of the F1-V and the G-V in KCNQ1+KCNE1 with a model that not only includes two state transitions for the VSDs, but also assumes independent VSD movement (Zaydman et al., 2014). Although constitutive currents are seen in the KCNQ1 charge reversal (E1R/R2E and E1R/R4E) mutants (which arrested VSDs in the intermediate and activated states, respectively), when these mutants are co-expressed with KCNE1, the E1R/R2E IKs currents are eliminated whereas the E1R/R4E IKs currents increase 10-fold. The absence of E1R/R2E IKs currents suggest that in the presence of KCNE1, the PD cannot open when VSDs are in the intermediate state but can open when 0−4 VSDs are in the activated state. Thus, the second fluorescence step is equated with VSD transitions and not PD opening. From these experiments intermediate-open (IO) states were omitted from the 30-state KCNQ1 model, to generate a model for IKs where at least one VSD must be activated for the PD to open regardless of the state of the other VSDs (Zaydman et al., 2014).

## VSD Mutants Prove Useful

Several groups have directly addressed the question of a requirement for all voltage sensors to move for the PD to open by using voltage sensor mutations to change the voltage-dependence of VSD movement, or prevent it altogether. R231W and L353K stabilize the channel in the open state and R243W stabilizes the channel in the closed state in the presence of KCNE1, and when mutant KCNQ1 subunits in KCNQ1 concatenated tetramers were co-expressed with KCNE1, a linear relationship between the shift in the voltage dependence of activation and the number of mutated subunits was observed (Meisel et al., 2012). This suggested that each subunit contributed incrementally to channel gating, and that much like KCNQ1 alone, the KCNQ1/KCNE1 channel complex could open when 0−4 VSDs were activated. Mutant cycle analysis of these constructs revealed very low values of inter-subunit free energy coupling values, which is more consistent with weak allosteric gating transitions than conformational changes comprising large and cooperative quaternary rearrangements, and supports the conclusion that a concerted cooperative activation step is not required. Consistent with the results from the R243W mutant in KCNQ1, macroscopic IKs currents were also seen in the depolarizing KCNE1 mutant, F57W, in one, two or four KCNE1 subunits concatenated with KCNQ1 subunits, and the voltage-dependence of activation was progressively depolarized as the number of F57W subunits in IKs complexes was increased. As well, decreased conductance levels were seen in both whole cell and single channel recordings with increasing numbers of mutated subunits (Westhoff et al., 2019), rather like that seen when R243W was expressed in three of four subunits (Meisel et al., 2012).

**FIGURE 3:**
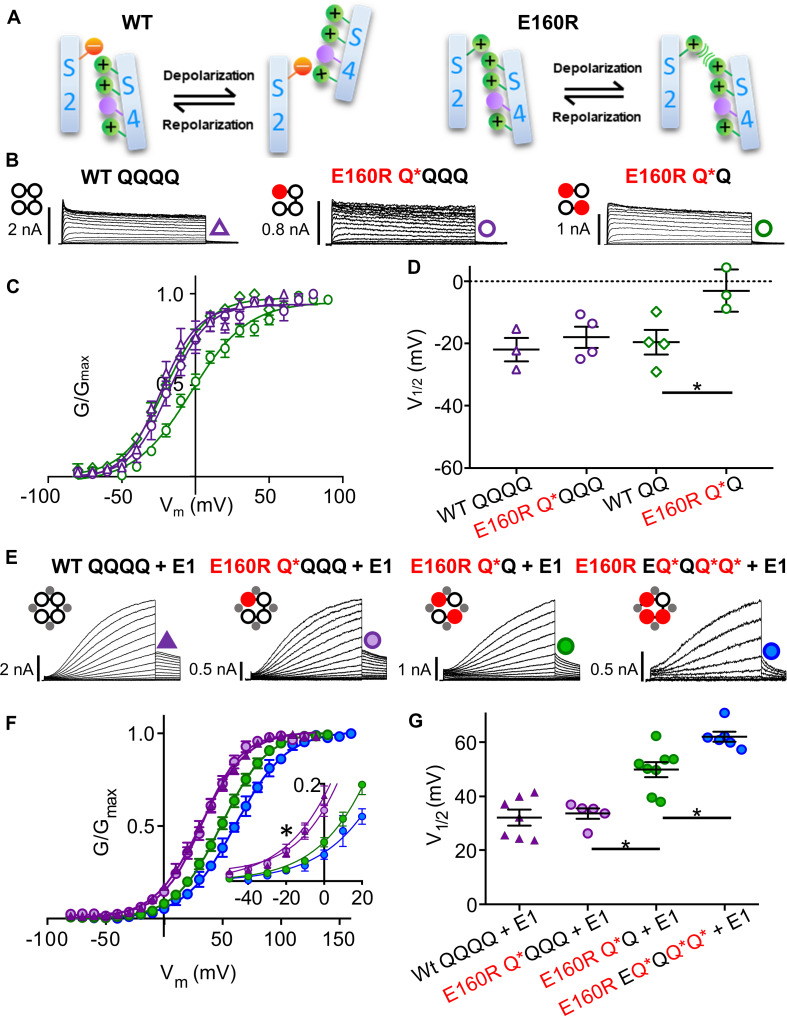
Functional currents from KCNQ1 and KCNQ1 + KCNE1 channel complexes with one, two or three E160R mutations **(A)** Cartoons representing possible charge interactions between WT S2 and S4 TMDs during activation and deactivation (left) and E160R mutants when VSD movement is impeded by charge repulsion (right). Channel topology configurations of WT Q1 (black) and E160R mutant (red) subunits, with E1 (gray) are shown adjacent to current traces **(B,E)**. Black lines represent tethers between subunits. Currents were obtained using the same protocol as in Figures 1B–D. In the absence of KCNE1. Currents **(B)**, mean G-V **(C)**, and summary V_1/2_ of activation **(D)** for KCNQ1 complexes containing zero (WT QQQQ: purple triangles; WT QQ: green diamonds), one (E160R Q*QQQ; purple circles) or two (E160R Q*Q; green circles) E160R mutant subunits (*n* = 3–4; *p < 0.05). **(E–G)** In the presence of KCNE1. Currents **(E)**, mean G-V **(F)** V_1/2_ of activation **(G)** for Q1 + E1 complexes containing zero (WT QQQQ; purple triangles), one (E160R Q*QQQ; purple circles), two (E160R Q*Q; green circles) or three (E160R EQ*QQ*Q*; blue circles) E160R subunits (*n* = 5–8; ^∗^*p* < 0.05). (**E**, Inset) Expanded view of G-V plots between –50 and +20 mV (^∗^*p* < 0.05). Figure adapted from Westhoff et al. (2019), Figures 1, 3.

A more extreme mutation is E160R, thought to prevent S4 translation altogether, rather than just shift the voltage dependence to very positive potentials (Zaydman et al., 2014). Recent experiments have conclusively established that VSDs containing this mutation are essentially held in a ground resting state by the electrostatic repulsion between E160R and R228 (Figure 3A; Westhoff et al., 2019): G229C residues which are buried at rest and can normally be modified by extracellular MTS reagents in wild-type subunits upon activation, cannot be labeled and so remain buried in E160R VSDs despite large depolarizations; G219C residues in E160R VSDs cannot be labeled upon depolarization by Alexa488 or do not undergo environmental change during applied depolarizations and so no change in fluorescence is detected; and E160R-containing subunits are correctly assembled into tetrameric channels as shown by introduced TEA sensitivity. Consistent with the R234W and F57W experiments, after introducing the E160R mutant in one or two, but not four concatenated KCNQ1 subunits, subjectively normal macroscopic IKs currents are observed (Figure 3B), although activation kinetics are changed as the G-V V_1/2_ is depolarized and the slope is decreased (Figures 3C,D). Similarly, in the presence of KCNE1, the E160R mutation in 1−3 subunits gives whole cell currents with depolarized V_1/2_s of activation, decreased activation delays (Figures 3E,F) and decreased conductance levels in both whole cell and single channel recordings as more E160R subunits are incorporated into tetramers (Westhoff et al., 2019). Taken together, these results all suggest that a final cooperative concerted voltage sensor transition is not required during the activation process for either KCNQ1 channels (Osteen et al., 2010; Westhoff et al., 2019) or complexes of KCNQ1 and KCNE1 (Meisel et al., 2012; Zaydman et al., 2014; Westhoff et al., 2019). The channel PD can open at rest, when a single voltage sensor reaches an intermediate activated state in KCNQ1 channels, and when it reaches the fully activated state in KCNQ1+KCNE1 channels. However, there are penalties incurred when less than four VSDs activate and these include a greater energy barrier to opening as suggested by the progressive depolarizing displacement of the V_1/2_ of the G-V, and reduction in the single channel conductance (Meisel et al., 2012; Westhoff et al., 2019).

## A Consensus Between Allosteric Models of KCNQ1 and IKs Activation Gating

A number of different non-cooperative allosteric models have been proposed for the activation gating and PD opening of KCNQ1 and KCNQ1+KCNE1 channel complexes. As noted earlier, models of only KCNQ1 (Osteen et al., 2010) and KCNQ1±KCNE1 (Zaydman et al., 2014; Westhoff et al., 2019) support the activation of single independent VSDs with allosteric coupling to opening of the PD (see below). These are analogous to activation gating models proposed for the related BK and HCN channels which suggest that with each successive VSD activation, the probability of PD opening increases multiplied by an allosteric factor “L” raised to the power of the number of activated subunits (Horrigan et al., 1999; Altomare et al., 2001; Flynn and Zagotta, 2018). In support of these non-concerted models, machine learning methodology applied to atomistic modeling of IKs is able to simulate most aspects of activation gating, including two-step voltage sensor displacement, I-V relationships and subconductance states – in this case determined by the pore energy profile (Ramasubramanian and Rudy, 2018). Modeling also demonstrates that restraining the movement of one, two and three VSDs, does not affect the ability of channels to conduct current in the manner demonstrated experimentally (Meisel et al., 2012; Westhoff et al., 2019), which supports non-concerted coupling between VSDs and the PD. In these simulations KCNQ1/KCNE1 was found to open to prominent subconductance levels. Subconductance was least when KCNQ1 lacked KCNE1 accessory subunits, and the addition of KCNE1 allowed access to progressively larger subconductance states, which effectively reproduced experimental recordings of IKs single channels from fixed channel complexes of differing numbers of KCNE1 subunits (Figure 1; Murray et al., 2016).

## Voltage Sensor Domain – Pore Domain (VSD−PD) Coupling

This topic has been well reviewed previously and we would encourage readers to consult representative reviews for a broader, more detailed historical coverage (Blunck and Batulan, 2012; Vardanyan and Pongs, 2012; Barros et al., 2019). Here, we will update VSD-PD coupling focused on KCNQ1, considering newer data, particularly from fluorescence (Zaydman et al., 2013; Barro-Soria et al., 2017; Hou et al., 2017, 2019, 2020), single channel recordings (Werry et al., 2013; Hou et al., 2017; Thompson et al., 2017; Westhoff et al., 2019) and recently published cryo-EM structures of KCNQ1 (Sun and MacKinnon, 2017, 2020).

## Electromechanical Coupling

**FIGURE 4:**
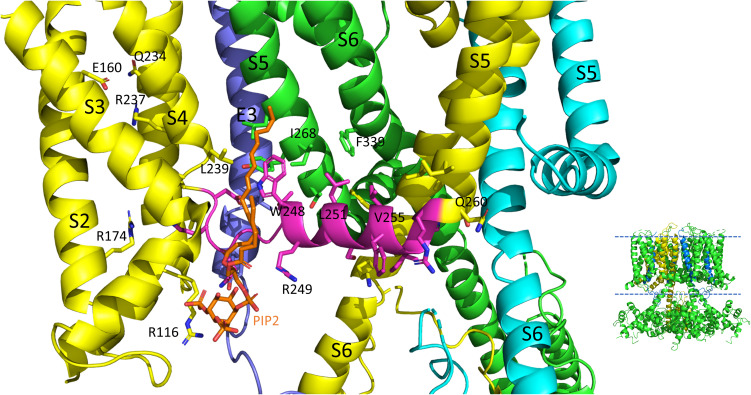
S4-S5L interactions of the PIP2 bound hKCNQ1/KCNE3/CaM structure. Each of three subunits of the tetramer is shown in a different color. The S4-S5L of the yellow subunit is shown in magenta for emphasis. Side chains of residues of the S4-S5L and residues within 4Å of these are shown, in addition to E160, R237 and Q234 in the yellow subunit to emphasize the activated state of the VSD. Only some of the more visible side chains are labeled but all residues appearing to interact with the linker include: R116, R174, I198, L239, H240, V241, Q260, E261, L262, and I263 (yellow subunit); E261, Y267, I268, and L271 (green subunit); none (cyan subunit); L75 and Y79 (KCNE3); PIP2. CaM and most of the intracellular portions (illustrated in inset to right), as well as the VSD of the cyan subunit, a third entire subunit and all but one KCNE3 (dark blue) have been removed for clarity. Image made from pdb 6V01 (Sun and MacKinnon, 2020) using PyMOL software (The PyMOL Molecular Graphics System, Version 2.2.3 Schrödinger, LLC).

The process of linking the movement of the VSD to pore opening is termed electromechanical coupling. In domain-swapped Kv channels this is accomplished through the S4-S5 linker (S4-S5L) and specific interactions with the C-terminal domain of S6 (Figure 4). Coupling of the VSD and PD can be disrupted by specific mutations or interventions in both of these regions in KCNQ1 and IKs (Choveau et al., 2011; Labro et al., 2011; Ma et al., 2011), and indeed residues in these two locations appear to co-evolve with one another in Kv channels (Lee et al., 2009). Our understanding of how these disparate portions of the channel work together to effect channel opening upon depolarization was greatly facilitated by the publication of the first crystal structure of a domain swapped Kv channel by the MacKinnon lab in Long et al. (2005b). The structure showed the helical S4-S5 linker sitting on a splayed C-terminal portion of the S6 domain. The diameter of the inner vestibule indicates that this is an open conformation of the PD, and as there are no closed-state structures of Kv channels, many studies have hypothesized ways in which S4-S5L can open the closed pore, playing the role of lever or bolt (Blunck and Batulan, 2012; Chowdhury and Chanda, 2012; Barros et al., 2019). In the first case the S4 must drag on the S4-S5L, which sits as a cuff at the helical bundle formed by the four S6 domains crossing one another, and cause the S5 domains to pull the S6 domains apart. In the second case the S4-S5L is theorized to act like a bolt to hold the S6 domains together to constrict the PD, akin to the ligand/receptor model (Choveau et al., 2012), where the removal of the bolt or ligand (S4-S5L) allows the S6 domain to passively open. The mechanism utilized depends on the intrinsic property of the pore, whether it is more stable in the closed state (*Shaker*, Kv1.2) or more stable in the open state (KCNQ1, HCN2) reviewed in Chowdhury and Chanda (2012), Vardanyan and Pongs (2012). A significant number of VSD and PD mutations give rise to a constitutive component of KCNQ1 current and form part of the evidence that places it in the latter group. In addition, KCNQ1 lacks the glycine hinge and the PVP motif in the bundle crossing (Seebohm et al., 2006; Labro and Snyders, 2012) found in several other Kv channel S6 domains which creates a kink in the helix to make up the narrowest region of intrinsically stable closed pores (Blunck and Batulan, 2012).

## Beyond the Mechanics, PIP2 Is Essential for VSD-PD Coupling

On top of the fundamental mechanical steps, the process of KCNQ1 channel opening is highly regulated. PIP2 depletion is known to decrease IKs channel currents (Loussouarn et al., 2003; Zhang et al., 2003; Park et al., 2005) but this depletion does not prevent S4 movement, as robust fluorescence recordings are still possible (Zaydman et al., 2013; Barro-Soria et al., 2017) and so it was concluded that the loss of PIP2 was uncoupling VSD movement from opening of the pore. Molecular dynamics simulation and other experimental results have mapped the binding site of PIP2 to residues in the S2-S3 and S4-S5L and the cytoplasmic end of the S6, placing PIP2 in a location in which it can interact with both the VSD and PD and thus affect VSD-PD coupling (Thomas et al., 2011; Zaydman et al., 2013; Eckey et al., 2014). In support of these data and conclusions, a cryo-EM structure with PIP2 bound (Figure 4) shows where the lipid is located near positive charges in the N-terminal domain (R116) and S2-S3L (R181, K183, R195, and K197) but also L239 in S4, Q244, W248 and R249 in the S4-S5L as well as K87 of KCNE3.

Mutations that prevent pore closure, and show instantaneous currents during step potential changes are either insensitive to PIP2 depletion (e.g., KCNQ1-L353K; Zaydman et al., 2013) or lose their voltage-dependent component (KCNQ1-I268A; Barro-Soria et al., 2017). An activated pore does appear to reduce some of the energy required to move the voltage sensor as long as coupling is intact. Not having to open the pore in the case of the uncoupled mutant V254M (middle of the S4-S5L, Figure 4) also appears to shift the F-V to slightly more negative potentials, though this was not the case with other uncoupled mutants closer to the PD (Hou et al., 2020).

**FIGURE 5:**
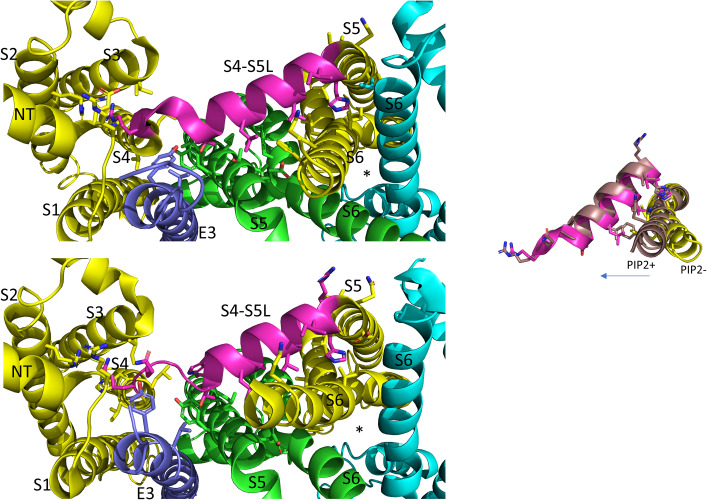
In the PIP2 bound state the KCNQ1 S4-S5L appears to have pivoted out and S6 has expanded into the space vacated. View is from below. Each of three subunits of the tetramer is shown in a different color. The S4-S5L of the yellow subunit is shown in magenta for emphasis. The PIP2-free structure of hKCNQ1/CaM/KCNE3 is on top (6V00) and the PIP2-bound structure is on the bottom (6V01). The asterisks indicate the location of the inner vestibule. Most of the intracellular portions and transmembrane regions of the other subunits have been removed for clarity. Inset: overlay of just the linker and the covalently linked S6 subunit, in PIP2 bound and unbound states highlighting changes related to PD opening. Arrow points in direction of expansion. Image made from pdbs 6V01 and 6V00 (Sun and MacKinnon, 2020) using PyMOL software (The PyMOL Molecular Graphics System, Version 2.2.3 Schrödinger, LLC).

Molecular dynamic simulations and an NMR structure have shown that KCNE1 sits in the lipid-filled cleft between two adjacent VSDs and the PD, probably not particularly dissimilar to where KCNE3 is found in cryo-EM structures (Figures 4, 5; Sun and MacKinnon, 2020), and based on this positioning is well placed to affect VSD-PD coupling. Consistent with this, mutations in the S4-S5L of KCNQ1 alone such as L251A, V255W, H258A and T247A, have produced similar changes to the activation waveform and G-V as those seen when KCNE1 is present in the complex (Labro et al., 2011). In the presence of KCNE1, the channel complex has also been shown to become more sensitive to PIP2 as a significantly lower concentration of exogenous PIP2 was required to prevent rundown upon excision of membrane patches.

**FIGURE 6:**
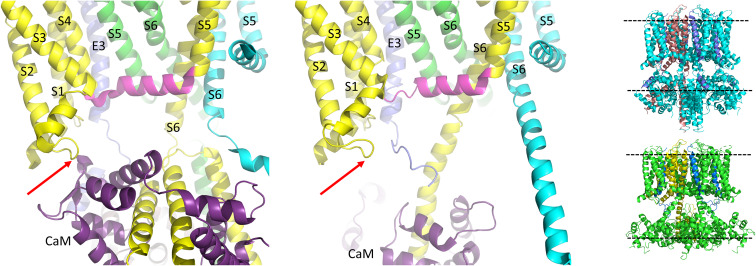
CaM unbinds from the S2-S3L in the PIP2 bound structure of hKCNQ1/KCNE3/CaM. Each of three subunits of the tetramer is shown in a different color. The S4-S5L of the yellow subunit is shown in magenta for emphasis. Only one CaM molecule is shown in the PIP2-free structure on the left (6V00) and the PIP2-bound structure in the middle panel (6V01) and most of the intracellular portions (illustrated in insets to right), as well as the VSD of the cyan subunit, a third entire subunit and all but one KCNE3 have been removed for clarity. Red arrow points to CaM interaction site on the S2-S3L. Image made from pdb 6V00 and 6V01 (Sun and MacKinnon, 2020) using PyMOL software (The PyMOL Molecular Graphics System, Version 2.2.3 Schrödinger, LLC).

In the last few years the published Cryo-EM structures of KCNQ1 (Sun and MacKinnon, 2017, 2020) have proven very useful in the interpretation of functional data. To date, all the published structures have activated VSDs (E160 is interacting with Q234 and R237, Figure 4) with the PD either uncoupled from the VSD due to a lack of PIP2, or a PIP2- containing structure in which the intracellular gate is open or opening but the filter is too narrow to coordinate a dehydrated potassium ion (Sun and MacKinnon, 2020). Whether there are more steps in the activation pathway that have not been transmitted to the selectivity filter or whether this is a feature of the KCNQ1 pore is at present unknown. The structure of human KCNQ1 with calmodulin (CaM) and KCNE3 created in the absence of PIP2 shows the S4-S5L to have a less structured N-terminal portion (Figure 5, upper panel) than the Kv1.2 S4-S5L, which is all helical. In addition, the linker itself is angled away from S6 which decreases the interaction between these two domains. In the presence of PIP2, several changes were noted and these include: the S6 domains are more splayed open, with the diameter at the putative S6 gate (S349) opening to >3.5 Å; the region after the S6 TMD becomes helical and makes continuous the S6 and HA domains; the interaction between the S2-S3L and CaM is lost and CaM is now reoriented 180 degrees toward the central axis of the channel complex. It is tempting to speculate that perhaps PIP2 is required in the coupling process to displace CaM so that the C-terminus can reorient itself for channel opening (Figures 5, 6).

Comparing the charge pairing, with respect to E160 in the VSD, it appears that the same state has been captured in all of the human KCNQ1 structures (Sun and MacKinnon, 2020), with the E160 residue interacting with both Q234 and R237, which would, depending upon the pore conformation correspond with AC (where AC represents a state where the VSD is in the activated state and the pore is closed) (Zaydman et al., 2014) in the case of 6V00 when PIP2 is absent, or activated-open (AO) perhaps in 6V01 when PIP2 is present. In the cryo-EM structures, rearrangement of the interactions of the S4-S5L show it shifting outward, making room for its covalently linked S6 to splay open (Figure 5). The linker no longer makes contact with the neighboring S6 at V355 a residue previously implicated in the coupling process (Choveau et al., 2011) and mutations all along the bottom of S6 have been shown to affect activation, changing voltage dependence and kinetics (Boulet et al., 2007), highlighting the importance of this region as the channel gate (Figure 5). How the channel might reorient as it returns through the intermediate closed and then to the resting state, and how KCNE1 might influence this process will have to await new structures being solved. Until then, experiments utilizing peptides (Choveau et al., 2011), state specific mutations and molecular dynamics will continue to be used to obtain further insight into pore opening processes (Zaydman and Cui, 2014; Hou et al., 2017, 2019, 2020).

## IKs Pore Openings Show Multiple Conductance Levels

Even in the absence of single channel recordings it was clear that the openings of IKs were different from most other Kv channels (Yang and Sigworth, 1998). Not only did early data from macropatches suggest a high frequency flicker behavior, but in multi-channel patches sub-conductance behavior was observed. What is the origin of subconductance behavior? It has been proposed that if the pore enters heteromeric states that are sub-conducting and a late step in activation were relatively slow, then sublevels would be evident at all membrane potentials (Chapman et al., 1997) as is observed in IKs (Werry et al., 2013) and mutant Kv1.2 channels (Chapman et al., 1997; Chapman and VanDongen, 2005). IKs activation/opening could be slow for many potential reasons: (1) The movement of S4 is slow due to charge paucity (3 arginines compared to 7 positive charges in *Shaker*); (2) KCNE1 causes steric hindrance to the late movements of the VSD (i.e. the F2 component, Nakajo and Kubo, 2014), or the S4-S5L as KCNE3 is in direct interaction with the linker in the Cryo-EM structures and data suggest that the location of KCNE1 will not be significantly different (Melman et al., 2001, 2002a, 2004; Barro-Soria et al., 2017); (3) the nature of the S4-S5L, in that it is not all helical but possesses a loop connecting to the S4 domain (Figure 5; Sun and MacKinnon, 2017, 2020); (4) the dependence on PIP2 binding for coupling; (5) a requirement for structural rearrangement of the C-terminal domain and CaM reorientation (Figure 6). Whether it is all or some of these reasons or even additional factors, IKs clearly opens very slowly with currents failing to saturate even on depolarization for 100 s (Pusch et al., 2000). In addition, as discussed earlier, IKs appears relatively unique in another respect, the ability of the pore to open when only an individual VSD in the tetramer has activated (Figure 3; Westhoff et al., 2019), creating another mechanism for subconductance behavior.

Multiple subconductance states and opening levels makes the assignment of a single conductance value for the IKs channel difficult, as noted by Yang and Sigworth, as conductance values will be dominated by the higher sub-conductance states due to the generally higher probability of occupancy of these states at potentials where single channel currents are measured (Yang and Sigworth, 1998). Initially, as was historically typical, single channel data were analyzed using the half-amplitude criterion to assign open levels (Werry et al., 2013), but subsequently sub-conductance levels for IKs have been assigned using a 3/2 rule that was derived from analysis of 26 types of ion channels for assigning multiple conductance states to channels (Pollard et al., 1994). From patches with a well-defined Gaussian distribution and clear peak for the main opening level (see all points histogram for EQ in Figure 1A), which did not appear to be affected by filtering, whether at 200 or 1000 Hz, the remaining sub-conductance states were calculated based on the 3/2 rule (Werry et al., 2013; Murray et al., 2016; Thompson et al., 2017). In support of a 3/2 division, longer lived subconductance occupancies were clearly observed at the predicted levels. Single channels showing prominent subconductance behavior have subsequently been obtained from LQT1 mutants of KCNQ1 (Eldstrom et al., 2015), different stoichiometric ratios of KCNE1 to KCNQ1 (Figure 1A; Murray et al., 2016), during exposure to cAMP or mefenamic acid (Thompson et al., 2017, 2018a; Wang et al., 2020), and as a result of individual VSD movement (Westhoff et al., 2019). All these studies observed that subconductance behavior tended to be more stable at the onset of activity upon depolarization when it is more likely that not all VSDs are activated. Single channels have also been recorded from KCNQ1 in the absence of KCNE1 (Figure 1B; Hou et al., 2017). In this situation conductance of KCNQ1 alone is too small to analyze for subconductance behavior, but in the absence of KCNE1 coupling appears to be fairly tight as the F-V and G-V all but overlap (Osteen et al., 2010), and thus subconductance openings might not be a frequent occurrence in KCNQ1 alone.

When VSDs are stabilized in the activated state via charge reversal between the S2 domain and the S4 domain, E160R/R237E (Hou et al., 2017) or through gain of function mutations (S209F; Werry et al., 2013), or phosphorylation-dependent enhanced activation (Thompson et al., 2017, 2018a), higher sub-conductance levels are more predominant, but in all these instances a fast flickering of channel behavior during bursts is seen (Figures 1A–C). This may reflect the transient occupancy of a closed state not in the activation pathway (Werry et al., 2013), or a pore block that is as yet undefined. For KCNQ1 alone, the fast flicker process is reduced in high Rb^+^ solutions, which may be part of the reason that these currents through KCNQ1 are bigger than with K^+^ solutions (Pusch et al., 2000). Once KCNQ1 pairs with KCNE1, the pore architecture appears to be different and Rb^+^ is no longer as permeable. This is also true of ML277-bound KCNQ1, and may be related to ML277 preventing inactivation of the homomer (Xu et al., 2015), as there is a strong link between inactivation and Rb^+^ permeability for KCNQ1 (Seebohm et al., 2003). Interestingly, IKs carrying the atrial fibrillation-linked mutation (AF) S140G in KCNQ1, has an increased Rb^+^ permeability compared with wild-type IKs (Peng et al., 2017). A potential mechanism is suggested by recently published structures where both the region of the S1 domain containing S140 and the N-terminal domain of KCNEs approach the N-terminal portion of the filter helix.

## Inactivation of KCNQ1

Unlike IKs which appears not to undergo inactivation, KCNQ1 alone has been suggested to undergo two inactivation processes. A fast inactivation phase is sometimes seen after the initial current activation, but is most clearly revealed and studied as a transient increase in conductance after membrane repolarization to potentials which exceed –50 mV (Pusch, 1998; Hou et al., 2017; Meisel et al., 2018). An additional voltage-dependent slow inactivation phase has also been reported primarily in mutant KCNQ1 channels including the LQTs-associated mutations S338W (S339 in hKCNQ1) and L273F (Gibor et al., 2007; Hou et al., 2017; Meisel et al., 2018). Although various molecular mechanisms for inactivation have been proposed, the two KCNQ1 inactivation processes are in contrast to the classical N-, C- and U-type mechanisms of inactivation.

Fast N-type inactivation, often referred to using the analogy of a “ball-and-chain” mechanism, was originally proposed for sodium channel inactivation, and later for potassium channels, and involves a charged amino-terminal intracellular peptide domain which swings in after channel activation and occludes the PD to prevent ion conduction (Armstrong et al., 1973; Hoshi et al., 1990, 1991; MacKinnon et al., 1993; Aldrich, 2001; Gebauer et al., 2004). In *Shaker* channels, a single independently acting inactivation particle has been found to be both necessary and sufficient for pore occlusion and prevention of ion conduction (MacKinnon et al., 1993; Zhou et al., 2001). However, the lack of a N-terminal ball structure in KCNQ1 suggests that the channel does not undergo N-type inactivation. C-type inactivation, on the other hand involves conformational changes in the selectivity filter in the outer pore region (Hoshi et al., 1990; Baukrowitz and Yellen, 1996; Cuello et al., 2010) with other hallmarks including inhibition by high external K^+^ (Lopez-Barneo et al., 1993), as in the case of hERG channels (Baukrowitz and Yellen, 1995; Wang et al., 1997). Whether these conformational changes in the selectivity filter result in pore constriction or subtle pore dilation is presently not understood. The lack of dependence on extracellular K^+^ for both the fast and slow inactivation processes, and the acceleration of slow inactivation in the presence of high external K^+^ (Meisel et al., 2018) suggests that KCNQ1 perhaps also does not undergo C-type inactivation (Pusch et al., 1998). Alternatively, it can be argued that KCNQ1 inactivation shares some similarities with C-type inactivation as slow inactivation has been shown to involve conformational changes in the outer carbonyl ring of the selectivity filter (Gibor et al., 2007). Discussed in greater detail later on, selectivity differences for Rb^+^ and K^+^ also differ between KCNQ1 which undergoes inactivation, and IKs which does not undergo inactivation (Pusch, 1998; Pusch et al., 2000; Seebohm et al., 2003). Lastly, U-type inactivation implicated in Kv1.5, Kv2.1, and Kv3.1 channels is the least understood of the potassium channel inactivation mechanisms (Klemic et al., 2001; Kurata et al., 2002; Kurata and Fedida, 2006; Cheng et al., 2010, 2011). The term U-type inactivation originates from the U-shaped inactivation-voltage curve which defines an inactivation process that is more complete at intermediate membrane voltages than at more positive potentials. The N-terminus and residues in the S3-S4 and S5-S6 linker have all been shown to play a role in U-type inactivation (Kurata et al., 2002; Cheng et al., 2010, 2011), but with fast inactivation only evident at particular times and potentials, again, U-type inactivation does not seem to underlie inactivation of KCNQ1 (Pusch et al., 1998).

To explain the initial fast inactivation in KCNQ1, a model which consisted of at least two distinct open states with voltage-independent transitions between the last open state and a non-conducting inactivated state was proposed (Pusch et al., 1998). KCNE1 was thought to either slow or prevent the transition to the last open state from which inactivation occurs. The presence of at least two open states was later independently confirmed using intracellular Na^+^ block. The first open state, accessed when the channels are briefly opened, displayed relative insensitivity to intracellular Na^+^ block. A stronger more sensitive block by Na^+^ was reported after the channel was depolarized for a longer interval, thereby driving the KCNQ1 channel into a second open state (Pusch et al., 2001). On the basis of at least two open states, a model which accounted for KCNQ1’s high Rb^+^/K^+^ conductance ratio was proposed as a rapid equilibrium process (flicker) between a flicker-open and flicker-closed state (Pusch et al., 2000). At high Rb^+^ concentrations, the flicker-open state was favored over the flicker-closed state with the flickering rate slowed. A positive correlation between the extent of inactivation and Rb^+^/K^+^ conductance ratio was later used to explain inactivation in terms of flicker states (Seebohm et al., 2003). Inactivation was proposed to result when the flicker rate between the second flicker-closed and second flicker-open state was faster than that for the first flicker-closed and first flicker-open state suggesting that the two open states had different Rb^+^/K^+^ conductance ratios. As the association of KCNE1 decreases the Rb^+^/K^+^ conductance ratio, inactivation is therefore also abolished.

A recent reinterpretation of inactivation in KCNQ1 channels, is based on an activation model with two open states, and is also cognizant of changes in Rb^+^/K^+^ conductance ratio (Hou et al., 2017). As each KCNQ1 channel contains four VSDs, the original 30 state full activation gating scheme discussed previously can be simplified into a six-state scheme where the VSD undergoes resting to intermediate, and then to activated transitions, while the PD can open when the VSD is in any state. In prior work, KCNQ1 was found to open initially into the IO state (where IO represents a state where the VSD is in the intermediate state and the pore is open) and then transition to an AO state (activated-open) (Zaydman et al., 2014). However, when the channel is in the AO state, VSD-pore coupling is less efficient and as such, pore opening requires higher voltages than in the IO state. Thus, when channels transition from the IO to the AO state in KCNQ1 during depolarizations to positive potentials, the total current will be reduced over the time spent depolarized, resulting in a process that imitates inactivation, and a “hook” in the tail current upon repolarization as channels return from AO to higher open probability IO states and subsequently close (Hou et al., 2017). Suppression of the IO and AO states by the KCNQ1 mutants F351A and S338F, unsurprisingly also eliminated inactivation.

This novel mechanism for inactivation, due to different mechanisms of VSD-pore coupling which occur in the IO and AO states, also explains why the co-assembly of KCNQ1 with KCNE1 abolishes inactivation. As mentioned previously, since co-assembly with KCNE1 eliminates the IO state, inactivation which arises from transitions between the IO and AO state are therefore also eliminated. As alluded to by the authors, though, this model likely does not account for the second slow inactivation phase seen in numerous KCNQ1 mutant channels. The L273F KCNQ1 mutant has been reported to exhibit both fast and slow inactivation (Gibor et al., 2007; Hou et al., 2017; Meisel et al., 2018) and the incorporation of the L273F mutant into increasing numbers of KCNQ1 subunits incrementally stabilizes a second inactivated state by progressively slowing down the inactivation recovery kinetics (Meisel et al., 2018). From this work Meisel and colleagues proposed that slow inactivation gating of mutant KCNQ1 channels likely occurs through an allosteric mechanism. Presently, it is not well understood if this slow inactivation is only seen in mutant KCNQ1 channels. If this slow inactivation is seen in WT KCNQ1 channels, how this allosteric mechanism fits into the presently understood mechanisms for KCNQ1 activation gating and fast inactivation also remains to be determined.

## Modulators of KCNQ1 and IKs

**FIGURE 7:**
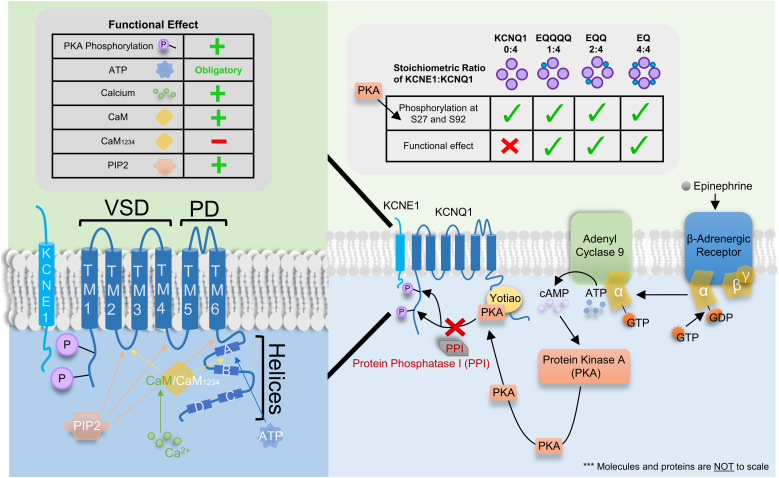
Binding site, signaling pathway and functional effect of various intracellular signaling molecules and co-factors on IKs. Left Table: Functional effect of protein kinase A (PKA) phosphorylation, ATP, Ca^2+^, CaM, CaM_1234_, and PIP2 on IKs where “+” and “–” denotes a stimulatory and inhibitory effect, respectively. “Obligatory” indicates ATP is required for channel conductance. Left panel: Cartoon of the single transmembrane β-subunit KCNE1 and the α-subunit KCNQ1. KCNQ1 consists of 6 transmembrane domains (TM1-4 and TM5-6 form the voltage sensor and pore domain, respectively) and 4 helices (A–D). Four KCNQ1 subunits come together to form the channel with 1-4 KCNE1 subunits. Binding sites for ATP, CaM/CaM_1234_ and PIP2 as well as sites of PKA phosphorylation are depicted using colored arrows (for binding sites) or directly on KCNQ1 (for PKA phosphorylation). Ca^2+^ is known to bind to CaM but not CaM_1234_ however, Ca^2+^ may modulate the channel by binding and interacting with other proteins and/or other locations on the IKs channel complex which are presently unknown. Right Table: The impact of KCNE1:KCNQ1 stoichiometry on the phosphorylation of residues S27 and S92 by PKA and the consequent functional effect. Checkmarks indicate PKA phosphorylation occurs or a functional effect is seen on the respective stoichiometrically fixed KCNE1:KCNQ1 complex (EQ, EQQ, EQQQQ, and KCNQ1). The “X” indicates a functional effect is not seen despite PKA phosphorylation of KCNQ1. Right panel: Signaling pathway for β-adrenergic enhancement of IKs current through PKA phosphorylation.

The kinetics of KCNQ1 are not only affected by co-assembly with KCNE1, but also by modulation induced by several intracellular signaling molecules and co-factors such as calmodulin (CaM), ATP, and protein kinase A (PKA). Figure 7 graphically summarizes the signaling pathway for β-adrenergic enhancement of IKs (right panel) as well as the binding sites (left lower panel) and impact of these intracellular proteins on IKs (top tables).

## CaM Modulates Current Amplitude and Assembly

One of the first pieces of evidence that calcium and potentially calcium binding proteins could modulate IKs was the sensitivity of the current to calcium in guinea pig cardiomyocytes (Tohse, 1990). Calmodulin (CaM), a highly conserved calcium binding protein has been recognized as a key modulator of various channels such as Cav1.2 and Nav1.5 all of which are involved in the regulation of the cardiac action potential (Sorensen et al., 2013). As mutations in CaM, KCNQ1 and KCNE1 have all been linked to LQTS, it is not surprising that CaM plays a diverse role in both IKs assembly and gating. Using metal affinity chromatography, CaM was first found to co-purify with a 6xHis-tagged C-terminus portion of KCNQ1 (Ghosh et al., 2006). Binding of CaM to the C-terminus portion of KCNQ1 was later confirmed using immunoprecipitation in both the presence and absence of Ca^2+^ (Shamgar et al., 2006) as well as structurally using x-ray crystallographic data (Dvir et al., 2014). This C-terminus structure was also further confirmed in the recent cryo-EM structure of KCNQ1 (Sun and MacKinnon, 2017). Specifically, CaM was found to bind to the two proximal cytosolic helices (helices A and B) in a 1:1 ratio of KCNQ1 to CaM. In addition to this C-terminal binding site, however, the cryo-EM structure also revealed a novel CaM binding site located at the S2-S3 loop of the VSD (Figure 6) not present in the original crystallographic structural representation which did not contain the transmembrane domains of the channel (Sachyani et al., 2014; Sun and MacKinnon, 2017, 2020). Thus, by interacting with both the VSDs at the S2-S3 loop and helices A and B (Figure 7, left panel), CaM was recently proposed to provide a potential link between the VSD and PD. In support of this hypothesis, PIP2, previously found to be critical in VSD-PD coupling has been shown to compete with CaM for binding at helix B at specifically residues K526 and K527 (Tobelaim et al., 2017). As such, CaM, much like PIP2, may also act to stabilize the channel in its open state.

Beyond this recent discovery that CaM may play a role in VSD-PD coupling, classically, CaM has more consistently been reported to be important for channel assembly (Ghosh et al., 2006; Shamgar et al., 2006; Sachyani et al., 2014). Point mutations in helix A (1375D) and helix B (V516D) which altered CaM binding have been shown to reduce protein expression and decrease current density suggesting that CaM may be required for proper channel assembly, folding and tetramerization (Haitin et al., 2008; Sachyani et al., 2014). In addition, KCNQ1 has also been reported to traffic to the cell surface in a process dependent on Ca^2+^ and CaM. Jiang et al. have shown that, following Angiotensin II type 1 receptor (AT1R)-mediated KCNQ1 trafficking, the channel may then combine with KCNE1 which in turn increases IKs current amplitude (Jiang et al., 2017). The Ca^2+^-insensitive CaM mutant, CaM_1234_, however, was shown to prevent AT1R stimulation from increasing IKs current density, suggesting that CaM not only plays a role in channel assembly and tetramerization but also in trafficking.

Electrophysiological studies have also provided consistent evidence supporting the important roles calcium and CaM play in enhancing both KCNQ1 and IKs current. Increasing intracellular Ca^2+^ concentrations has been found to enhance KCNQ1 current amplitude (Shamgar et al., 2006). Consistent with this, chelation of Ca^2+^ by BAPTA has been reported to decrease KCNQ1 current amplitude (Ghosh et al., 2006) with the previously reported increase in current amplitude following administration of intracellular Ca^2+^ (Shamgar et al., 2006) possibly explained by the ability of CaM to relieve KCNQ1 inactivation in a Ca^2+^ dependent manner (Ghosh et al., 2006). In the presence of KCNE1, the role of CaM in enhancing current amplitude is preserved. Both the co-assembly with CaM_1234_ (Sachyani et al., 2014) and bath application of the CaM antagonist W7 (Shamgar et al., 2006) have been reported to decrease IKs current amplitude. Interestingly, CaM_12_ but not CaM_34_ was also shown to decrease IKs current amplitude, suggesting that not only is CaM able to modulate IKs, but its modulation is highly dependent on Ca^2+^, and specifically where Ca^2+^ binds in the KCNE1-KCNQ1-CaM complex (Sachyani et al., 2014). From a cell signaling point of view, the stimulatory action of CaM has been shown to be modulated by calcium/calmodulin-dependent protein kinase II (CaMKII). Intracellular application of the CaMKII inhibitor, autocamtide-2 as well as another inhibitor, KN-93, reduced IKs current amplitude. KN-93 preincubation was also able to prevent any stimulatory action of intracellular CaM administration suggesting that CaMKII may act to turn off CaM enhancement of IKs (Xie et al., 2015). However, more studies to confirm and uncover the full signaling pathway of CaM mediated IKs enhancement and channel assembly are still needed.

## ATP Is Essential for Channel Opening

Several studies have demonstrated that the removal of ATP decreases or completely abolishes IKs activity, with current rescued by the reintroduction of ATP (Loussouarn et al., 2003). Using photo-crosslinking, the phosphates of ATP were suggested to electrostatically interact with a cluster of basic residues (R380, K393, and R397), whereas the nucleoside moieties of ATP were suggested to interact with hydrophobic aromatic residues such as W379 in the helix A to helix B region downstream from the S6 segment (Li et al., 2013; Figure 7, left panel). Functional studies have indicated that, although both W379S and the double mutant R380S/R397W abolish KCNQ1 currents, F-V signals are still present and are identical to that of WT KCNQ1, which indicates that ATP binding is not required for VSD movement. Moreover, in a prior study, PIP2, known to be essential for VSD-PD coupling, was found to hyperpolarize the F-V curve of a constitutively open KCNQ1 mutant, L353K (Zaydman et al., 2013). This shift was abolished in the absence of PIP2, but removal of ATP sites in the background of L353K did not eliminate the displacement of the F-V curve, suggesting that ATP is perhaps not involved in VSD-PD coupling (Li et al., 2013). These results suggest that ATP plays a role in PD opening through interactions with the KCNQ1 C-terminus, but other ATP binding sites may exist which could in the future reveal a role for ATP in either VSD movement and/or VSD-PD coupling.

## β-Adrenergic Enhancement of Current Through PKA Phosphorylation

Under stressful conditions, circulating sympathetic hormones such as epinephrine bind to and activate G-protein coupled β-adrenergic receptors leading to the release of the α-subunit of the G-protein (Terrenoire et al., 2005; Figure 7, right panel). This α-subunit in turn activates adenyl cyclase 9 which leads to an increase in the intracellular levels of cAMP. The binding of cAMP then activates protein kinase A (PKA), which phosphorylates the C-terminus of KCNQ1 in the presence of Yotiao (Marx et al., 2002). Specifically, PKA phosphorylation has previously been described to occur at residues S27 and S92 (Marx et al., 2002; Lopes et al., 2007; Lundby et al., 2013). Although KCNQ1 alone has been found to be largely unresponsive to the functional transduction of PKA phosphorylation (Kurokawa et al., 2003), the phosphorylation of the N-terminus of KCNQ1 in the presence of KCNE1 produces a left-shift in the voltage dependence of activation, a slowing of deactivation kinetics and an increase in current amplitude (Dilly et al., 2004; Terrenoire et al., 2005). Together these changes in kinetics increase repolarizing current and shorten the cardiac action potential to allow sufficient time for ventricular filling. PKA-mediated phosphorylation is later removed by protein phosphatase 1. cAMP is degraded by the cAMP-specific phosphodiesterase, PDE4D3.

Using linked constructs which fix the KCNE1:KCNQ1 stoichiometry at different ratios (Murray et al., 2016), the hyperpolarizing shift in the voltage dependence of activation following cAMP exposure was progressively increased with increasingly saturated IKs complexes: the more KCNE1 in the complex, the greater the hyperpolarization, which showed that not only is the action of cAMP on IKs stoichiometrically dependent, but also that only one KCNE1 subunit is required for a basal response of the IKs complex to cAMP (Thompson et al., 2018b). In support of this latter finding, shortening of the first latency to opening in the presence of cAMP was seen in IKs constructs with KCNE1:KCNQ1 ratios of 1:4 and 2:4, similar to that seen in fully saturated IKs complexes. Using a phosphomimetic KCNQ1 mutant, this shortening of the first latency following cAMP treatment seen in WT channels was reduced but not completely abolished in the mutant S27D channel, indicating that this residue is only partly responsible for the shortening of first latency seen post-cAMP (Thompson et al., 2018a). Although phosphorylation of residue S27 plays a partial role in shortening of the first latency, it appears to be largely responsible for cAMP mediated changes in open probability. The double phosphomimic mutant S27D/S92D on the other hand completely abolished any changes in first latency, single-channel conductance and subconductance distribution as a result of cAMP exposure. Mechanistically, these effects of phosphorylation are largely mediated by enhancement of VSD activation, allowing the channel to open more frequently, quicker, and to higher sublevels, as mutant channels with fixed-activated VSDs showed little further response to cAMP (Thompson et al., 2017).

## Conclusion

The KCNQ1 channel current is highly modulated by co-assembly with a wide range of accessory molecules and proteins, some of which have been reviewed here. Of most significance is the modulation of KCNQ1 by its β-subunit, KCNE1. Through the use of linked constructs which fix the ratio of KCNE1 to KCNQ1, the stoichiometry of association has been definitively shown to be variable between 1:4 to 4:4. Despite this, discussions surrounding the stoichiometry of KCNE1 and KCNQ1 remain relevant, not only as the KCNE1:KCNQ1 ratio in humans is presently unknown, but also because the stoichiometry of the IKs channel complex influences the efficacy of various therapeutic drugs and the action of accessory molecules such as CaM and ATP, and β-adrenergic enhancement of IKs via PKA phosphorylation.

The co-assembly of KCNE1 with KCNQ1, apart from changing the response to accessory molecules, also alters channel kinetic properties such as activation and deactivation, increasing conductance, and eliminating both the fast and slow inactivation seen in KCNQ1 channels. Recent experiments have suggested that these changes are mediated without fundamental changes in the allosteric coupling of voltage sensors to the pore activation gate, and although allosteric gating of KCNQ1 channels is generally accepted, much work still remains to be done in order to fully understand gating of KCNQ1 in association with its accessory subunits.

Molecular dynamics and docking simulations in combination with the recent cryo-EM structures of KCNQ1+CaM and KCNQ1+KCNE3+CaM have given many structure-function insights into the binding of various accessory molecules − notably, PIP2 which is essential for VSD-PD coupling, and CaM which plays a wide range of roles including in channel assembly and as an activator. Still, the exact binding location of the full KCNE1 subunit within the KCNQ1 tetramer remains uncertain. Given the many actions of KCNE1 and the physiological importance of the IKs channel, cryo-EM structures of KCNQ1+KCNE1 in both the presence and, where possible, the absence of essential accessory molecules such as CaM, PIP2 and ATP is probably the next major step needed to gain a more robust understanding of how KCNE1 is able to dramatically modulate the channel properties of KCNQ1.

## Author Contributions

All authors contributed equally to the writing and editing of this manuscript.

## Conflict of Interest

The authors declare that the research was conducted in the absence of any commercial or financial relationships that could be construed as a potential conflict of interest.

## Abbreviations

G-V: conductance-voltage relationship
IKs: delayed cardiac rectifier K^+^ current
I-V: current-voltage relationship
*k*: slope factor
LQTS: long QT syndrome
V_1/2_: voltage at half-maximal activation or inactivation
VCF: voltage clamp fluorimetry
VSD: voltage sensor domain
WT: wild type.
